# Oxidative Stress Induced by Excess of Adiposity Is Related to a Downregulation of Hepatic SIRT6 Expression in Obese Individuals

**DOI:** 10.1155/2018/6256052

**Published:** 2018-12-23

**Authors:** Marcos C. Carreira, Andrea G. Izquierdo, Maria Amil, Felipe F. Casanueva, Ana B. Crujeiras

**Affiliations:** ^1^Laboratory of Molecular and Cellular Endocrinology, Instituto de Investigacion Sanitaria de Santiago (IDIS), Complejo Hospitalario Universitario de Santiago (CHUS), Santiago de Compostela, Spain; ^2^CIBER de Fisiopatologia de la Obesidad y Nutricion (CIBERobn), Instituto Salud Carlos III, Madrid, Spain; ^3^Laboratory of Epigenomics in Endocrinology and Nutrition, Epigenomics Unit, Instituto de Investigacion Sanitaria de Santiago (IDIS), Complejo Hospitalario Universitario de Santiago (CHUS/SERGAS), Santiago de Compostela, Spain; ^4^Universidad de Santiago de Compostela (USC), Santiago de Compostela, Spain

## Abstract

Sirt6 is a member of the sirtuin family involved in physiological and pathological processes including aging, cancer, obesity, diabetes, and energy metabolism. This study is aimed at evaluating the relationship between liver *SIRT6* gene expression and the oxidative stress network depending on adiposity levels in Zucker rats, an animal model of metabolic syndrome. We observed that liver-specific *SIRT6* expression is reduced in an in vivo model of spontaneous obesity and metabolic syndrome. We also observed that *SIRT6* expression in the liver is positively associated with *SIRT1* and *GST-M2* expressions, two proteins involved in antioxidant protection pathways and inversely related to body weight and plasmatic oxidative status. Interestingly, the *SIRT6* expression is upregulated after energy restriction-induced weight loss concomitantly with an improvement in oxidative stress markers. These results suggest that *SIRT6* may be a potential therapeutic target for the treatment of obesity and associated metabolic disorders, such as liver disease.

## 1. Introduction

During the last years, numerous evidences suggested the oxidative stress as a key factor involved in the development of obesity and its comorbidities [1]. The oxidative stress in obesity is induced by an excessive generation and accumulation of reactive oxygen species (ROS) in different cellular structures due to the expansion of the adipose tissue and inefficiency in the energy metabolism leading to cellular damage [2, 3]. The metabolic syndrome associated with obesity identifies subjects who have an increase in morbidity and mortality and is correlated with the development of several pathologies that affect different organs such as the liver and the progression from steatosis to nonalcoholic steatohepatitis and hepatocarcinogenesis in which oxidative stress appears to be involved [4].

Sirtuins are a family of NAD^+^-dependent protein deacetylases and ADP-ribosyltransferases with an important role in regulating the life span, aging, and cancer as well as energy metabolism and obesity and its metabolic associated disorders [5] and have therefore been proposed as a possible target for future therapies against these diseases.

Seven highly conserved family members of sirtuins have been identified (Sirt1-Sirt7) in mammals [6]. A number of studies revealed that Sirt1 has several beneficial effects on metabolic cell control and enhances the ability of cells to cope with oxidative stress [7, 8]. However, relatively little is known about the other sirtuins Sirt2 to Sirt7 being suggested that seven sirtuins may have redundant or similar cellular functions with Sirt1 [9]. In this regard, the nucleus-specific Sirt6 level is involved in obesity and diabetes [10, 11]. Aging and overnutrition lead to decreased Sirt6 level resulting in alterations of glucose and lipid metabolism [10]. Deletion of Sirt6 in mice resulted in lethal hypoglycemia [9, 12, 13]. On the other hand, overexpression of Sirt6 improves blood lipid profiles in animals fed with high-fat diets [12]. Liver expression of Sirt6 is induced by caloric restriction and suppressed in diseases associated with lipid accumulation in the liver [12]. Hepatic-specific deletion of sirt6 resulted to triglyceride accumulation and liver steatosis [14]. In addition, adipose tissue-specific ablation of Sirt6 resulted in increased blood glucose, hepatic steatosis, and diet-induced obesity [10, 13]. Sirt6 levels are reduced in the adipose tissue of murine models of obesity and increased in the adipose tissue of humans with weight loss [15, 16].

Therefore, the aim of this study was to evaluate the hepatic gene expression of *SIRT6* and its relationship with the oxidative stress network depending on adiposity levels in Zucker rats, an animal model of metabolic syndrome.

## 2. Experimental Procedures

### 2.1. Animals

Male lean (Fa/fa; *n* = 10) and obese (fa/fa; *n* = 10) rats of the Zucker strain, 8 weeks old purchased from Charles River Laboratories (Barcelona, Spain), were maintained in controlled conditions of temperature, humidity, and illumination (12 h controlled photoperiod). They were allowed to acclimatize for 1 week on arrival. All rats had free access to water and standard laboratory diet (SAFE; Panlab, Spain), with 5.5% lipid, 23% protein, and 70% carbohydrate content. Body weight and food and water intake were measured during the experimental period. Finally (22 weeks), animals were euthanized and decapitated, and the livers and blood were obtained, immediately frozen on dry ice, and kept at −80°C until analysis. All animal experiments and procedures involved in this study were approved by the Ethical Committee at the University of Santiago de Compostela, in accordance with the European Union Normative for the use of experimental animals.

In the experimental weight loss protocol, fatty rats (*n* = 30) were randomly divided into three subgroups: an energy-restriction group (ER; *n* = 10), an exercise group (EX; *n* = 10), and an energy restriction plus exercise group (EREX; *n* = 10). These fatty rats were individually housed for 1 week, and their individual food intake was weighed and recorded. Then, the rats in the ER and the EREX groups were fed a diet 30% less in quantity than their individual food intake during 4 weeks (based on the weight of food).

Animals from the EX and the EREX groups were placed on a monitored rodent treadmill (Treadmill system 303401-R-04/C, TSE-Systems, Inc., Chesterfield, MO, USA) for 10 min/day and increased progressively in intensity from 10 m/min to 20 m/min during 1 week for familiarization. After that, animals were placed on the treadmill for 30 min/day at 20 m/min, 7 days per week for 4 weeks.

### 2.2. Body Composition

Body composition studies were performed every 2 weeks using a nuclear magnetic resonance imaging (MRI) system (Whole Body Composition Analyser, EchoMRI, Echo Medical Systems, USA).

### 2.3. RNA Extraction and Quantitative RT-PCR

Total RNA extraction from the liver was performed using Trizol (Invitrogen) according to the manufacturer's recommendations. The RNA (500 ng) was retrotranscribed into cDNA using the High Capacity cDNA Reverse Transcription Kit (Applied Biosystems, USA). The expression of the genes of interest was studied using TaqMan real-time PCR in Step One Plus system (Applied Biosystems, USA) using specific primers and probes obtained from inventoried TaqMan Gene Expression Assays (Applied Biosystems, USA) for SIRT6, SIRT1, and GST-M2 genes. All reactions were performed using the following cycling parameters: 50°C for 2 min, 95°C for 10 min, followed by 40 cycles of 95°C for 15 s, 60°C for 1 minute. For data analysis, the RNA level of the gene of interest was normalized using the *β*-actin values, according to the 2^-ΔΔCt^ method.

### 2.4. Oxidative Stress Blood Analysis

Plasmatic malondialdehyde (MDA) and total antioxidant capacity (TAC) were evaluated using commercially available colorimetric assay kits (OXIS International, Portland, OR, USA).

### 2.5. Statistical Analysis

The normal distribution was explored with the Kolmogorov-Smirnov test and the Shapiro-Wilk test. Because oxidative stress markers and gene expression levels were not normally distributed, the Mann–Whitney *U* test was applied to study the differences between obese and lean rats. The fold change in gene expression was calculated using the 2^-ΔΔCt^ relative quantitation method according to the manufacturer's guidelines (Applied Biosystems, USA) and reporting the data as the geometric mean (standard error of the mean, SEM). A *p* value < 0.05 was considered to be statistically significant, and a *p* value ≤ 0.1 was considered to be a trend for significance. The potential association between oxidative stress biomarkers and gene expression levels was evaluated using the Spearman rank correlation coefficient. Statistical analysis was performed by SPSS 15.0 software (SPSS Inc., USA) for Windows XP (Microsoft, USA) and GraphPad Prism 6.01 software (GraphPad Software Inc., USA).

## 3. Results

### 3.1. Characteristics of the Experimental Animal at 22 Weeks Old

We fed rats a standard diet while monitoring body weight gain and body composition. At the end of the experiment, the obese rats (fa/fa) showed higher body weight and consequently higher fat mass (9×) as well as lower free fat mass than their lean littermates (Fa/fa) (Table 1). In addition, plasma levels of oxidative stress biomarkers as MDA and TAC at the end of the experimental period were lower in lean than in obese phenotype (Table 1).

### 3.2. Hepatic Gene Expression of SIRT6, SIRT1, and GST-M2

**Figure 1:**
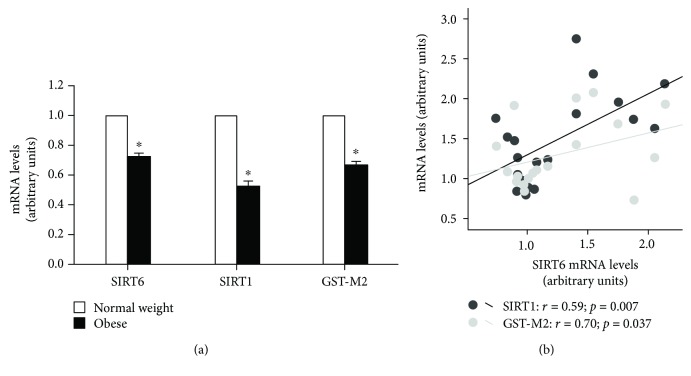
Liver expression of SIRT6, SIRT1, and GST-M2 genes in lean or obese rats (a). Data are represented as the mean ± standard error of the mean (SEM). Statistically significant differences compared with control-lean counterparts ^∗^ *p* < 0.05 vs. normal weight group. Association between SIRT6 mRNA levels with SIRT1 or GST-M2 genes in all animal taking together (b). SIRT1 (*r* = 0.59; *p* = 0.007), GST-M2 (*r* = 0.70; *p* = 0.037).

Obese rats showed a marked decrease in the hepatic gene expression of *SIRT6* (30%) and *SIRT1* (50%). These results in *SIRT6* and *SIRT1* gene expressions were also observed in an animal model of diet-induced obesity (DIO; Supplementary Figure 1). In addition, the hepatic gene expression of *GST-M2*, the antioxidant enzyme glutathione-S-transferase Mu2, was also reduced (30%) (Figure 1(a)).

Because *SIRT1* and *GST-M2* are two proteins with proven involvement in the antioxidant protection pathway, we performed an association study between *SIRT6* expression and *SIRT1* and *GST-M2* expression in livers from all rats taken together. Interestingly, the hepatic *SIRT6* mRNA levels were positively associated with the gene expression of *SIRT 1* (*r* = 0.59; *p* = 0.007) and *GST-M2* (*r* = 0.70; *p* = 0.037) (Figure 1(b)).

### 3.3. Association of *SIRT6* Gene Expression with Body Weight, MDA, and TAC

**Figure 2:**
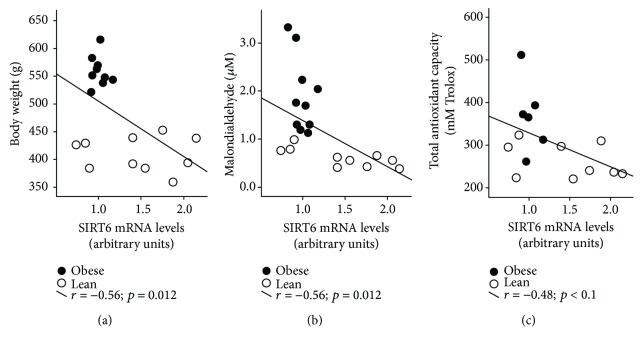
Association of SIRT6 gene expression with body weight (a), MDA (b), and TAC (c).

In accordance with the involvement of *SIRT6* in the regulation of oxidative stress process [10] and its association with *SIRT1* and *GST-M2* expression levels, we reasoned that the gene expression of *SIRT6* at the hepatic level should be correlated with body weight as well as with systemic markers of oxidative stress. In fact, increased hepatic *SIRT6* expression was associated with lower body weight (Figure 2(a)), lower plasma MDA levels (Figure 2(b)), and lower plasma TAC (Figure 2(c)).

### 3.4. Weight Loss, Systemic Oxidative Stress, and Hepatic Gene Expression

**Figure 3:**
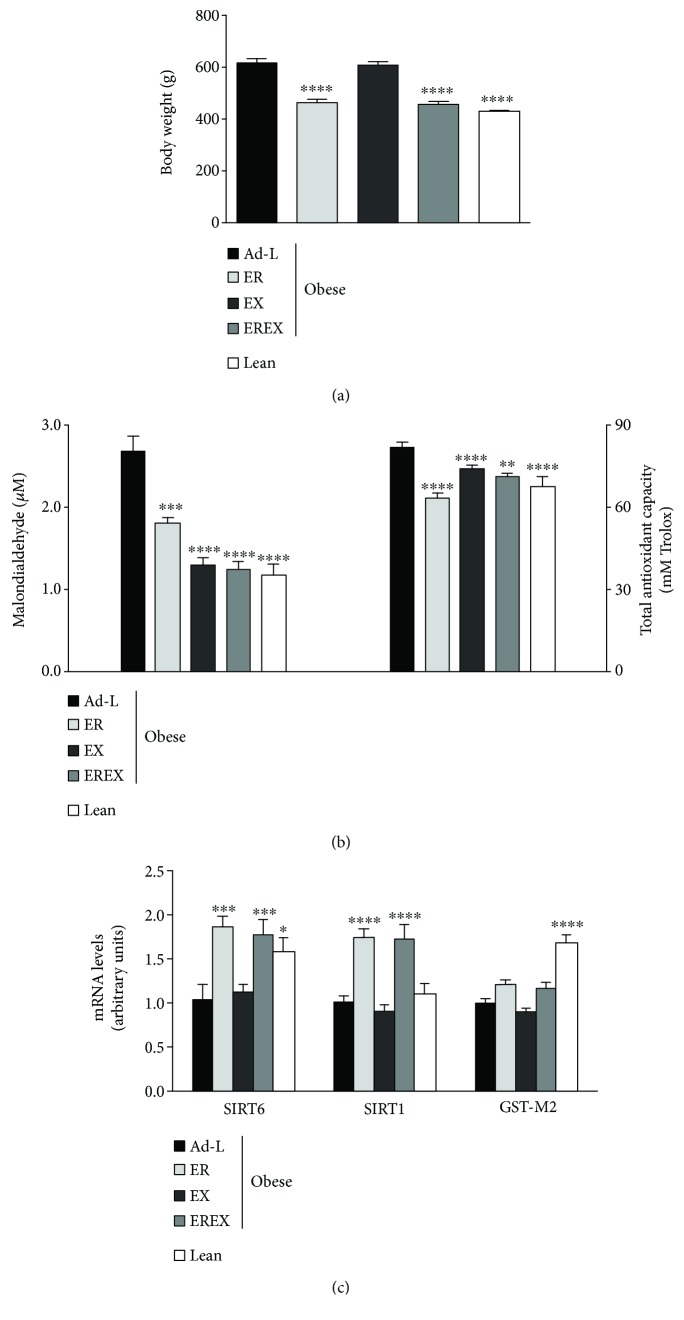
Effect of 4 weeks of weight loss interventions on body weight (a), serum oxidative stress biomarkers (b), and hepatic gene expression (c). Data are represented as mean and SEM, *n* = 6 − 10 animals/group. ^∗^ *p* < 0.05 vs. the Ad-L group, ^∗∗^ *p* < 0.01 vs. the Ad-L group, ^∗∗∗^ *p* < 0.001 vs. the Ad-L group, ^∗∗∗∗^ *p* < 0.0001 vs. the Ad-L group. Ad-L: ad libitum group; ER: energy-restriction group; EX: exercise group; EREX: energy restriction plus exercise group.

After the 4 weeks of weight loss treatments, the ER and EREX groups exhibited 26% less body weight (Figure 3(a)) than the Ad-L control group and similar to the lean control animals. No differences were observed between both groups or between the EX and the Ad-L group in body weight. According to the body weight loss data, the ER and EREX groups showed a significant reduction in the circulating levels of MDA and TAC (Figure 3(b)). Interestingly, in the EX group, despite not producing a reduction in body weight, it showed a reduction in the circulating levels of MAD and TAC similar to the effects observed for the EREX group (Figure 3(b)). Then, we investigated the effect of the weight loss interventions on hepatic gene expression of sirtuins and GST-M2. According to the body weight loss data, the ER and EREX groups but not the EX group showed a significant increase in SIRT6 and SIRT1 gene expressions (Figure 3(c)). However, the expression of GST-M2 showed no significant variations after the interventions (Figure 3(c)).

## 4. Discussion

This work shows that the oxidative stress induced by excess of adiposity is related to a downregulation of hepatic *SIRT6* expression in obese individuals. After weight loss induced by energy restriction, the hepatic *SIRT6* expression increases, concomitantly with an improvement in oxidative stress markers. Therefore, these results suggest that the potential role of SIRT6 in the protection against oxidative stress damage could be a therapeutic target to treat the damage caused by the association between obesity and oxidative stress.

Sirtuins play an important regulatory role in energy metabolism and they may be a potential therapeutic target for obesity and associated pathologies [5]. Among the sirtuin family members, sirt1 is the most well-studied sirtuin and it has been implicated in the protection against cellular oxidative stress, and it plays an important role in metabolic pathway regulation, specifically acting in adipocytes as an inhibitor of adipogenesis. Additionally, the expression of *SIRT1* is modulated by energy restriction in association with improvements in oxidative stress [7]. In this line, *SIRT6* was recently discovered as a relevant player in the predisposition to age-associated diseases [17]. The activity of *SIRT6* is reduced in obesity and diabetes and its hepatic-specific ablation increases liver steatosis onset [10]. However, the study of *SIRT6* is still very fresh [10]. In this work, we showed a downregulation of *SIRT6* in the liver of obese rats compared with their lean littermates.

The liver is a key metabolic organ controlling the overall lipid metabolism in response to hormonal and nutritional stimuli received and one of the organs most affected by excessive intake of carbohydrates or fat leading to metabolic pathologies associated with obesity. Several studies highlighted that obesity strongly contributes to the transition of nonalcoholic fatty liver disease (NAFLD) to nonalcoholic steatohepatitis (NASH) and hepatocellular carcinoma (HCC) [18, 19]. The absence of *SIRT6* increases the expression of genes responsible for hepatic long-chain fatty acid uptake and reduced expression of genes for *β*-oxidation leading to accumulation of triglycerides and fatty liver disease and hepatic steatosis [10]. Additionally, the participation of ROS in liver disease has been suggested [20]. Moreover, it was observed that genes related to oxidative stress regulation are overexpressed in early stages of HCC [21]. In this regard, obesity produces various metabolic alterations that contribute very actively to the general oxidative balance, creating the basis for the development of diseases such as diabetes, hypertension, cardiovascular disease, and cancer, among others. According to the literature, the major contributors to systemic oxidative stress in obesity are hyperglycemia, increased muscle activity to support weight gain, high lipid levels in different tissues, chronic inflammation, low antioxidant defenses, endothelial ROS production, and hyperleptinemia [1]. Oxidative stress in obesity is a systematic problem that can be reduced by improving antioxidant defenses through fat reduction, physical activity or exercise, dietary restriction, surgical intervention, or antioxidant therapies which, based on the results showed in this work, may include *SIRT6*.

In accordance with a potential association between the expression of *SIRT6* with oxidative stress, we observed a correlation between *SIRT1* and *GST-M2*, both genes that codify proteins involved in the protection against oxidative stress [7, 22], which were also downregulated in the liver of obese fa/fa rats. These results suggest a dysregulation in the antioxidant defenses that promote the oxidative stress characteristic of obesity [23] which was confirmed by the high circulating levels of MDA and TAC.

The connection between oxidative stress, energy restriction, and sirtuin activity has been shown in the literature. The energy restriction reduces the cellular levels of NADH by increasing the NAD+/NADH ratio and causing an increase in Sirt2 activity [24]. As in the case of Sirt2, Sirt6 activity is also influenced by energy restriction. Prolonged restriction results in increased activity of Sirt6 at the brain, muscle, white adipose tissue, and liver levels [12, 13]. In addition, Sirt6 is also a mediator of the effects induced by energy restriction. *SIRT6* suppression decreases life extension activated by energy restriction, and *SIRT6* overexpression shows reduced body weight, increased metabolism, and reduced serum levels of insulin, glucose, cholesterol, and several adipokines [13, 25].

In this sense, the data obtained in this study show that body weight loss is associated with an increase in hepatic *SIRT6* expression and a reduction in systematic oxidative stress biomarkers in a similar way to the well-studied *SIRT1*. According to the important role of Sirt6 in the liver related to lipidic and glucose metabolism [10], these effects are observed in models of caloric restriction; however, physical exercise does not seem to have any influence on hepatic SIRT6 expression, although exercise has a potent reducing effect of oxidative stress at the systemic level. This suggests that the exercise model produces a decrease in systemic oxidative stress similar to the energy restriction model but probably through a different mechanism in which the skeletal muscle may be involved. All these data suggest that *SIRT6* acts similarly to *SIRT1* and may play a key role in regulating energy metabolism and defense against oxidative stress.

In conclusion, the results of the current work evidenced that *SIRT6* gene expression shows similar pattern of *SIRT1* gene expression, the most-studied sirtuin member, in the context of relationship with excess body weight and the regulation of oxidative stress. It supports the idea of a prominent role for *SIRT6* as a potential therapeutic target for the treatment of obesity and associated disease, particularly liver disease.

## Figures and Tables

**Table 1:** Characteristics of the experimental animal at 22 weeks old.

	Obese (*n* = 10)	Lean (*n* = 10)	*p* value
Body weight (g)	559 ± 28	410 ± 30	<0.001
Fat mass (g)	213 ± 11	24 ± 6	<0.001
Free fat mass (g)	261 ± 49	303 ± 19	0.021
MDA (*μ*M)	1.75 ± 0.64	0.61 ± 0.19	0.001
TAC (mM Trolox)	436 ± 191	265 ± 40	0.020

Data are represented as the mean ± standard error of the mean (SEM). *P* value shows statistically significant differences compared with the control-lean group. MDA: malondialdehyde; TAC: total antioxidant capacity.

## Data Availability

The data used to support the findings of this study are available from the corresponding author upon request.

## Abbreviations

ANOVA: Analysis of variance
HCC: Hepatocellular carcinoma
HFD: High-fat diet
GST-M2: Glutathione-S-transferase Mu2
MDA: Plasmatic malondialdehyde
MRI: Magnetic resonance imaging
NAFLD: Nonalcoholic fatty liver disease
NASH: Nonalcoholic steatohepatitis
ROS: Reactive oxygen species
SIRT: Sirtuin
TAC: Total antioxidant capacity.

## Supplementary Materials

Supplementary Materials Supplementary Figure 1: liver expression of SIRT6 and SIRT1 in lean or diet-induced obesity (DIO) Sprague Dawley rats (A). Data are represented as the mean ± standard error of the mean (SEM). Statistically significant differences compared with control-lean counterparts ^∗^ *p* < 0.05 vs. lean group.
