# Supplementary figures and images for: Oxidative Stress Induced by Excess of Adiposity Is Related to a Downregulation of Hepatic SIRT6 Expression in Obese Individuals
Source: Oxid Med Cell Longev. 2018 Dec 23;2018:6256052. doi: 10.1155/2018/6256052 (PMC6317113; doi:10.1155/2018/6256052)

## Slide 1
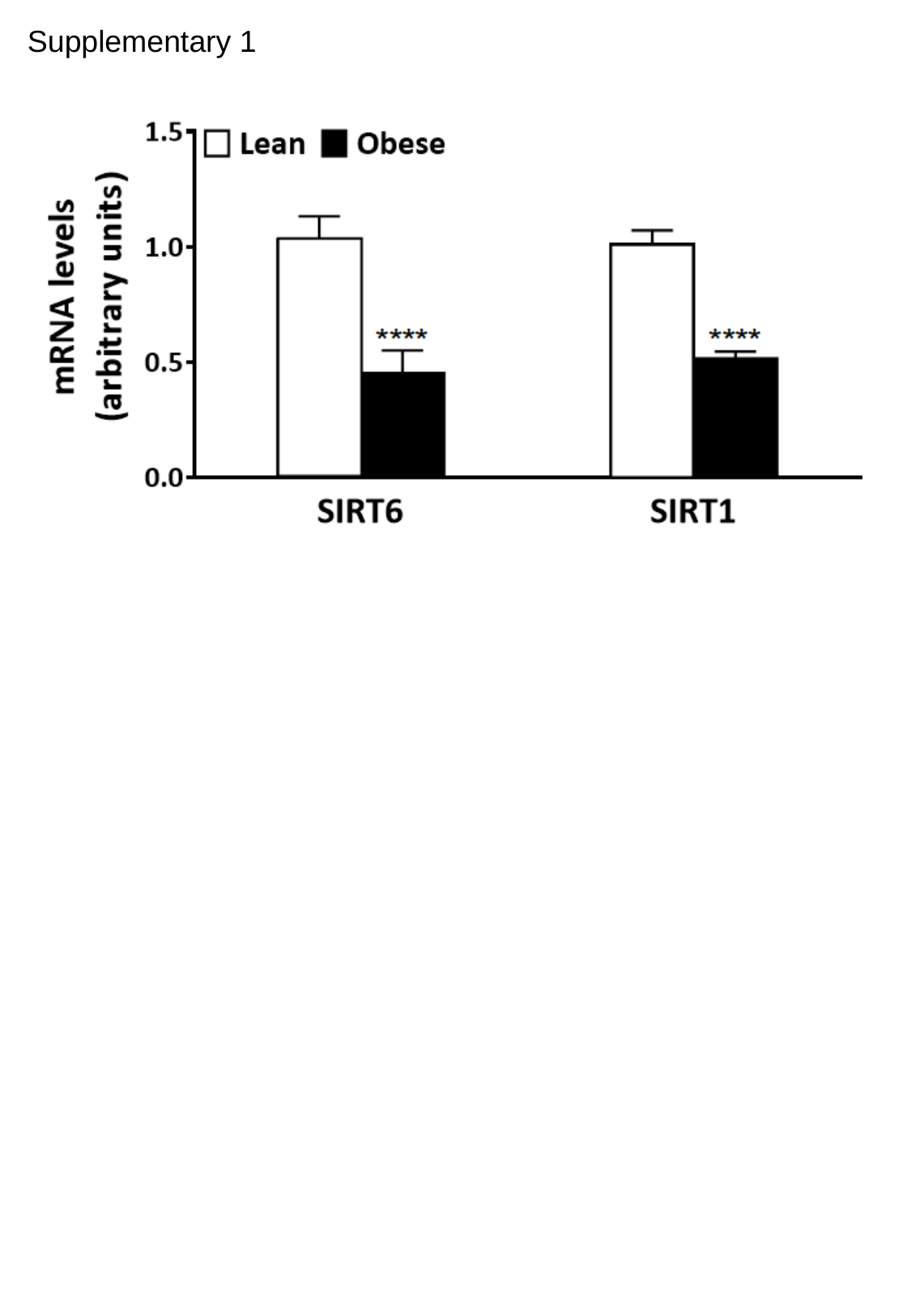

Supplementary 1

Supplement: Supplementary Materials — Supplementary Figure 1: liver expression of SIRT6 and SIRT1 in lean or diet-induced obesity (DIO) Sprague Dawley rats (A). Data are represented as the mean ± standard error of the mean (SEM). Statistically significant differences compared with control-lean counterparts ∗ p < 0.05 vs. lean group. [file 6256052.f1.pptx]
